# Reducing preventable adverse events in obstetrics by improving interprofessional communication skills – Results of an intervention study

**DOI:** 10.1186/s12884-022-05304-8

**Published:** 2023-01-23

**Authors:** Beate Hüner, Christina Derksen, Martina Schmiedhofer, Sonia Lippke, Sandra Riedmüller, Wolfgang Janni, Frank Reister, Christoph Scholz

**Affiliations:** 1 University Hospital Ulm, Department of Gynecology and Obstetrics, Prittwitzstr. 43, 89075 Ulm, Germany; 2 Jacobs University Bremen, Department of Psychology & Methods, Campus Ring 1, 28759 Bremen, Germany; 3 Muenchen Klinik, Department of Gynecology and Obstetrics, Muenchen, Germany

**Keywords:** Adverse events, Preventable adverse events, Obstetrics, Communication, Patient safety

## Abstract

**Background:**

Progress in medicine involves the structured analysis and communication of errors. Comparability between the individual disciplines is only possible to a limited extent and obstetrics plays a special role: the expectation of a self-determined and joyful event meets with possibly serious complications in highly complex care situations. This must be managed by an interdisciplinary team with an increasingly condensed workload. Adverse events cannot be completely controlled. However, taking controllable risk factors into account and with a focused communication a reduction of preventable adverse events is possible. In the present study, the effect of interprofessional team training on preventable adverse events in an obstetric department was investigated.

**Methods:**

The training consisted of a 4-h interdisciplinary training session based on psychological theories. Preventable adverse events were defined in six categories according to potential patterns of causation. 2,865 case records of a refence year (2018) and 2,846 case records of the year after the intervention (2020) were retrospectively evaluated. To determine the communication training effect, the identified preventable adverse events of 2018 and 2020 were compared according to categories and analyzed for obstetrically relevant controllable and uncontrollable risk factors. Questionnaires were used to identify improvements in self-reported perceptions and behaviors.

**Results:**

The results show that preventable adverse events in obstetrics were significantly reduced after the intervention compared to the reference year before the intervention (13.35% in the year 2018 vs. 8.83% in 2020, *p* < 0.005). Moreover, obstetrically controllable risk factors show a significant reduction in the year after the communication training. The questionnaires revealed an increase in perceived patient safety (t(28) = 4.09, *p* < .001), perceived communication behavior (t(30) = -2.95, *p* = .006), and self-efficacy to cope with difficult situations (t(28) = -2.64, *p* = .013).

**Conclusions:**

This study shows that the communication training was able to reduce preventable adverse events and thus increase patient safety. In the future, regular trainings should be implemented alongside medical emergency trainings in obstetrics to improve patient safety. Additionally, this leads to the strengthening of human factors and ultimately also to the prevention of second victims. Further research should follow up implementing active control groups and a randomized-controlled trail study design.

**Trial registration:**

The study was approved by the Ethics Committee of University Hospital  (protocol code 114/19-FSt/Sta, date of approval 29 May 2019), study registration: NCT03855735.

## Background

Since the publication of the report “To err is human” in the year 2000, the readiness for structured analysis of adverse events (AEs) in medicine has been increasing [1]. AEs are defined as treatment outcomes that are below the currently expected medical standard and result in temporary or permanent harm to patients [2].

The incidence of AEs in hospitalized patients ranges from 3 to 17%. Up to 50% of AEs are classified as preventable [3, 4]. As early as the 1980s, the Harvard Medical Practice Study showed that 4% of patients in a New York hospital were harmed by AEs. As a result, human and organizational factors became the focus of risk management, as these factors, together with insufficient medical knowledge, are seen as triggers of preventable adverse events (pAEs) that can be reduced through interventions [5, 6].

Intra- and interprofessional communication of health care workers (HCW) is a significant human element in health care. Faulty communication behavior is exacerbated by rapid team changes, high workloads, and lack of mutual respect. Lack of communication training and awareness of its importance leads to loss of essential information and compromises patient safety [7, 8]. PAEs can be attributed to inadequate communication in up to 70% of cases [9]. Error analyses in surgical departments showed that causal factors for adverse events were 65% due to human factors and only 4% due to technical problems [3].

PAEs also occur in obstetrics. In a study in Sweden 12.2% adverse events were detected of which 73.7% were assessed as preventable [10]. The special feature of obstetrics as a medical discipline lies in the coincidence of unpredictable emergencies, as well as the simultaneous responsibility for the expectant mother and the unborn child. However, the general expectation is that the birth of a child is a joyful and unencumbered event, and oftentimes possible complications are ignored [11]. This challenge must be met by an interdisciplinary team facing frequently changing staff, shift work, and different levels of competence. The consequences of pAEs in obstetrics are far-reaching and affect not only the future family but also the obstetric team involved. The obstetric team usually must deal with the possible medico-legal consequences of an incident in the long term and often suffers from the psychological burden of being "second victims" themselves [12, 13]. In obstetrics, too, feelings of guilt, anxiety, frustration, and self-doubt are reported as frequent consequences of pAEs [11]. The consequences are on the individual side with personal suffering, costs for the institution due to fines or compensation payments up to the loss of a job or an employee, resp., and thus further work intensification. It may even accelerate susceptibility to errors [14].

A reduction in pAEs can be achieved by improving interdisciplinary communication in the HCW team in obstetrics [15]. In a review of communication and teamworking trainings in obstetrics, an improvement in professional and empathic communication was observed in different programs. In 56%, communication was trained as the main construct and in 44%, communication was integrated into a broad teamwork training, including crew resource management or emergency simulation approaches. Most teamwork approaches have a clear communication component, as the allocation of resources and effective management of obstetric teams can only be achieved through effective interdisciplinary communication. Of the 71 studies that were included in the review, most focused on technical aspects of communication, e.g., effective handovers. 16 studies also reported clinical outcomes of communication, e.g., behavior in emergency simulation. Only three studies investigated teamwork in relation to the distal outcome of patient safety and medical outcomes, e.g., measured by the Adverse Outcome Index (AOI), of which only two showed moderate effects on patient safety [16]. This result can be partly explained by the fact that the preventability of AEs cannot be determined in a standardized way and often only serious "sentinel events" are reported.

An explicit definition and monitoring system for the risk factors for pAEs is needed to reduce the occurrence of pAEs [12]. This was done in an exemplary approach within the project TeamBaby. The research approach was developed for identifying pAEs, which could be used to evaluate a communication intervention. An interdisciplinary communication training for medical staff in obstetrics was designed and implemented. It focused on increasing patient safety through improved interprofessional communication of the obstetric team. These measures aimed to promote effective communication to reduce pAEs. To evaluate the effectiveness of the measures, categories of different pAEs after implementation of the TeamBaby communication training (year 2020) were compared to a reference year before the intervention (year 2018). The research hypothesis was that the frequency of pAEs would decrease in the year following the implementation of the training [15].

## Methods

The present study was conducted as part of the research project TeamBaby - Safe, digitally supported communication in obstetrics (Clinical Trials gov. Identifier: NCT03855735) . The study aims to improve interprofessional communication as well as that with expectant mothers and thus increase patient safety in obstetrics. The project is funded by the Innovation Fund (project no. 01VSF18023) of the Federal Joint Committee (G-BA) . Details of the research project have been published elsewhere [15].

### Evaluation of adverse and preventable adverse events

In the first phase of the project, pAEs were defined with the help of a list of criteria based on international research results as well as in project meetings with participants from different professions [17–19]. A list of 56 criteria (Appendix 1) classified as undesirable was finalized [12]. Of these, 30 events related to the physical condition of the mothers, 11 to the condition of the newborns, 12 events were assigned to interventional care, and 3 to the organizational area [17].

Based on the defined list of criteria, data were extracted from the obstetric documentation system of the University Hospital Ulm, Department of Gynecology and Obstetrics, Perinatal Center Level 1 , by clinical documentation assistants. All available data sources were used, such as the hospital's birth documentation (i.s.h.med system), the obstetric viewpoint system, and handwritten birth documentation. Pregnancies with less than 36 weeks gestation were excluded; multiple births were counted as one case.

Of a total of 3,351 births in the calendar year 2018 (reference year), 2,865 case records met the inclusion criteria, which were retrospectively evaluated for AEs according to the criteria of the previously defined list. The determination of preventability was independently assessed by an interprofessional team of three physicians and midwives based on the complete case analysis. Outcomes were classified into six defined categories for pAEs according to potential patterns of causation: 1) peripartum treatment delay (e.g., delayed intervention at birth, delayed intervention for postpartum hemorrhage), 2) diagnostic error (e.g., misdiagnosis of fetal birth position), 3) inadequate birth position, 4) organizational errors (e.g., lack of training, lack of documentation of birth progress), 5) inadequate fetal monitoring (e.g., fetal heart rate/maternal heart rate confusion on cardiotocography (CTG) or misinterpretation of the CTG, near-sudden infant death), and 6) medication errors. Each case could also be assigned to more than one category. In addition, the pAEs were evaluated according to obstetrically relevant risk factors and 13 risk factors, which include controllable and uncontrollable factors, were extracted [12].

For the present study, using the same criteria, the pAEs and risk factors in the obstetric data records of the calendar year 2020 after communication training in the same clinic were evaluated. From a total of 3,302 births, 2,846 case records were extracted analogously to the exclusion criteria mentioned above. To determine the communication training effect, the identified pAEs of 2018 and 2020 were compared according to categories and risk factors.

### TeamBaby communication training

The TeamBaby safe communication trainings were delivered by a company that specializes in patient safety training in obstetrics. The trainings were conducted by a midwife and an anesthesiologist experienced in obstetrics [20]. The training modules were developed in close cooperation with the project team consisting of psychologists, public health scientists, sociologists, and obstetricians. The training was theoretically based on health behavior change models and communication theories. The training sessions conducted lasted four hours and included individual behavioral planning for the application of what was learned.

The training content included mental models of "good" birth, a film to underline the importance of communication in crisis situations, as well as exercises in closed-loop communication, speaking-up, structured handover including ISBAR [16] and taking the perspective of the other professional group or mothers. Pocket cards as reminders as well as bi-weekly micro-teaching units via an online tool were developed for a sustainable consolidation of the contents. *N* = 65 staff members were trained in a total of six face-to-face training sessions, *N* = 33 of them doctors, *N* = 31 midwives, and one project site staff member. As the total workforce consists of approximately 70 healthcare workers, 93% could be trained. Additionally, *N* = 4 midwives who had not been trained before took part in the microteaching sessions. A detailed overview of the training content, exercises, and goals as well as the behavior planning intervention and microteaching have been published elsewhere [21].

### Evaluation of the self-reported data to evaluate the trainings

Questionnaires were used before and after the trainings to identify improvements in self-reported perceptions and behaviors. Perceived patient safety, operationalized as triggers for pAEs, communication behavior and self-efficacy to implement good communication was measured. For this purpose, scales with 7 items were used for perceived patient safety (Cronbach’s α = 0.73 at baseline/0.86 post-intervention) and communication behavior (Cronbach’s α = 0.86/0.88). Self-efficacy was measured through general self-efficacy (1 item) and coping with difficult situations (by 4 items; Cronbach’s α = 0.66/0.54). *N* = 32 HCW answered the questionnaire at both time points with codes allowing to match the pre-and post-intervention data sets. The evaluation was done with paired sample t-tests as well as the non-parametric Wilcoxon test.

The initial reactions of the participants and their acceptance of the training were captured with a feasibility questionnaire. The questionnaire asked about the healthcare professionals’ experience of the training in terms of the setting (5 items, Cronbach’s α = 0.63), satisfaction with the trainees (2 items, Spearman Rho = 0.63), training content (9 items, Cronbach’s α = 0.89), benefits of the training (8 items, Cronbach’s α = 0.59) and general acceptance (3 items, Cronbach’s α = 0.84). All responses were given on a four-point smiley scale (1 – two negative smileys to 4 – two positive smileys). Additional comments could be added to an open-ended question. Means and standard deviations are reported descriptively.

## Results

### Evaluation of the study sample

In the baseline characteristics, the sample collected in the year 2018 and 2020 differed statistically significantly in age (mean 31.2 vs. 31.7 years), body mass index (BMI) (25.06 vs. 25.41), and mode of delivery (Table 1).

**Table 1:** Baseline characteristics of women giving birth in 2018 or 2020 (after exclusion criteria)

	**2018**	**2020**	**p-value**
Total	2,865	2,846	
Maternal age (years), mean (SD)	31.25 (5.54)	31.72 (5.66)	< 0.001
Gestational age (weeks), mean (SD)	39.08 (1.31)	39.05 (1.30)	
Primiparous	47.6%	45.4%	
Multiparous (≥ 2)	52.4%	54.6%	
Body mass index (kg/m^2^)	25.06	25.41	< 0.007
**Birth mode (%):**			
Vaginal delivery	67.3%	61.9%	> 0.001
Caesarean section planned	11.6%	13.5%	> 0.001
Caesarean section unplanned	12.4%	14.2%	> 0.001
Instrumental vaginal delivery	8.7%	10.4%	> 0.001

In 2020, there were more caesarean sections (planned and unplanned) and more instrumental vaginal deliveries. The samples did not differ in the other baseline data regarding primiparous, multiparous and gestational age.

### Evaluation of institutionally tracked adverse events and preventable adverse events before and after communication training

After applying the list of criteria for AEs, 659 AEs were identified from 2,865 evaluated case data in the calendar year 2018 indicating 23.00%. In the calendar year 2020, 827 AEs were identified from 2,846 evaluated case data indicating 29.06%. This results in a significant difference in the number of AEs between the years 2018 and 2020 (*p *< 0.01).

In 2018, an AE was classified as preventable in 88 cases (3.07% of all evaluated data sets, 13.35% of the adverse events). In the calendar year 2020, AEs were classified as preventable in a total of 73 cases (2.57% of all evaluated data sets, 8.83% of the adverse events). This is a descriptive difference as expected. However, this difference in the number of pAEs proportionate to all births according to exclusion criteria was not significant (*p* < 0.248).

**Fig. 1:**
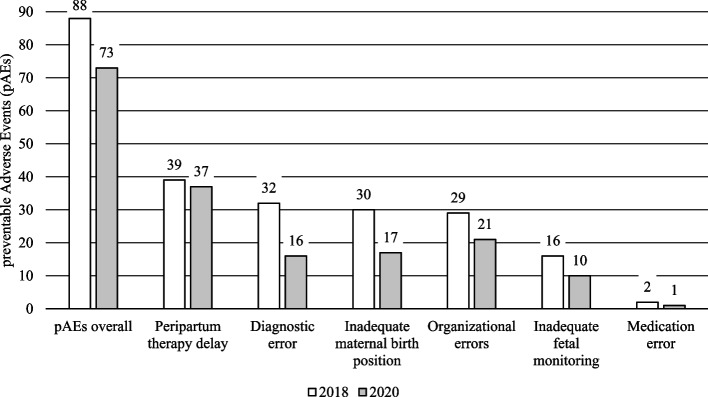
Preventable adverse events (pAEs) overall and categories before and after the communication training

In contrast, the pAEs in relation to the AEs were reduced from 13.35% in 2018 to 8.83% in 2020. This means a statistically significant reduction (*p* < 0.005) in pAEs of 33.9% after communication training proportionate to the AEs (Table 2, Fig. 1).

**Table 2:** Preventable adverse events (pAEs) overall and categories before and after the communication training proportionately to Adverse Events (AEs)

**pAE**	**2018**		**2020**		**Difference**	**p-value**
	Total	Percentage	Total	Percentage		pAEs proportionately to AEs
pAEs overall	88	13.35%	73	8.83%	-33.90%	0.005^a^
Peripartum therapy delay	39	44.32%	37	50.68%	14.37%	0.209^a^
Diagnostic error	32	36.36%	16	21.92%	-39.73%	0.002^a^
Inadequate maternal birth position	30	34.09%	17	23.29%	-31.69%	0.006^a^
Organizational errors	29	32.95%	21	28.77%	-12.71%	0.49^a^
Inadequate fetal monitoring	16	18.18%	10	13.70%	-24.66%	0.75^a^
Medication error	2	2.27%	1	1.37%	-39.73%	0.588^b^

*a* Pearson-Chi Quadrat, *b* Fisher Test

### Evaluation of the individual categories of avoidable adverse events before and after communication training

In 2018, the most common cause of pAEs was found to be a peripartum therapy delay in 39 cases (44.32%), followed by diagnostic errors in 32 cases (36.36%). Such pAEs due to an inadequate birth position, especially the supine position, occurred in 30 cases (34.09%). PAEs due to organizational errors occurred in 29 cases (44.32%). Inadequate fetal monitoring was the cause of pAEs in 16 cases (18.18%). Medication errors occurred in 2 cases (2.27%).

After the communication training, the categories of AEs classified as preventable were also evaluated in the year 2020. Peripartum therapy delay was again the most common with 37 cases (50.68%). A diagnostic error was found in 16 cases (21.92%). The inadequate birth position was the cause of a pAE in 17 cases (23.29%). In 21 cases (28.77%) there was an organizational error and in 10 cases (13.7%) inadequate fetal monitoring was the cause of a pAE. Another pAE was due to a medication error (1.37%). There was a statistically significant reduction in the categories of diagnostic error (*p* < 0.002) and inadequate birth position (*p* < 0.006). No significant statistical difference was found in other categories (Table 2).

### Comparison of risk factors in cases with adverse events before and after communication training

After applying the defined list of criteria for AEs, 659 cases with AEs were extracted in the year 2018, and 827 cases with AEs in the year 2020. In these case data, 13 obstetrically relevant risk factors were identified (Table 3). Comparing 2018 and 2020, there are no significant differences in the risk factors primiparous, multiparous, premature rupture of membranes, maternal age > 35 years, condition after caesarean section, pre-eclampsia, and diabetes. The risk factors of being on call duty, induction of labor, obesity, supine position at birth, and fetal macrosomia (LGA) occurred significantly less in the AEs cases in 2020 compared to 2018.

**Table 3:** Risk factors in the extracted AEs

Risk factors	2018 (*n* = 659)	2020 (*n* = 827)	p-value
Primiparous	426 (65%)	530 (64%)	0.824
Multiparous (defined as two births or more)	233 (35%)	289 (35%)	0.869
On-call duty (12 h shift, 6 pm-6 am)	406 (62%)	383 (46%)	< 0.001*
Induction of labor (IOL)	413 (63%)	458 (55%)	0.005*
Missed date of birth	267 (41%)	414 (50%)	< 0.001*
Obesity	153 (23%)	142 (17%)	0.004*
Premature rupture of membranes (PROM)	179 (27%)	205 (25%)	0.299
Back position at birth	225 (34%)	143 (17%)	< 0.001*
Maternal age > 35	193 (29%)	233 (28%)	0.637
Condition after caesarean section (CS)	132 (20%)	136 (16%)	0.74
Preeclampsia	44 (7%)	47 (6%)	0.427
Large for gestational age (LGA)	18 (3%)	58 (7%)	< 0.001*
Diabetes	96 (15%)	122 (15%)	0.92

p-value: Pearson-Chi Quadrat, * significant at p < *.01*

A possible interpretation of these results can be made by differentiating the risk factors into controllable and uncontrollable. The risk factors that do not show a significant difference are those that cannot be controlled, such as age or condition after a caesarean section. The risk factors that can be controlled are those such as birth position or induction of labor, which show a significant reduction in the year after the communication training. These differences can therefore be interpreted as a likely effect of the communication training on controllable risk factors.

### Evaluation of the self-reported data on interprofessional communication training

**Fig. 2:**
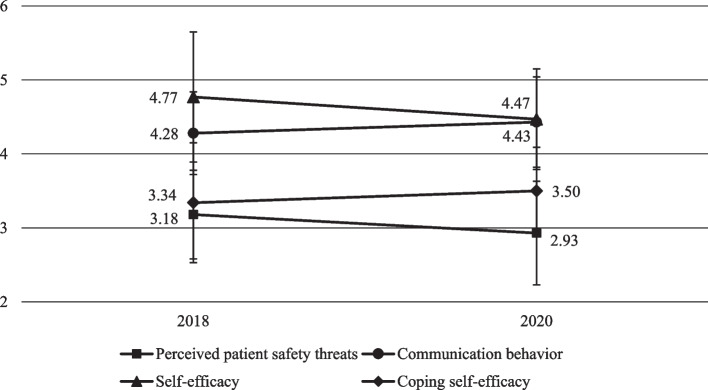
Evaluation of questionnaire data before and after the communication training (mean and standard errors)

The analysis of the data revealed an increase in perceived patient safety (t(28) = 4.09, *p* < 0.001), perceived communication behavior (t(30) = -2.95, *p *= 0.006), and self-efficacy to cope with difficult situations (t(28) = -2.64, *p* = 0.013). General self-efficacy decreased slightly descriptively, but only with a marginally significant effect(t(28) = 1.97, *p* = 0.059; Fig. 2). The results were replicated with the non-parametric Wilcoxon tests for all variables, with significant results for perceived patient safety (Z = -3.35, *p* < 0.001), communication behavior (Z = -2.6, *p* = 0.009) and coping self-efficacy (Z = -2.32, *p* = 0.021), but not action self-efficacy (Z = -1.9, *p* = 0.058).

59 HCW answered the feasibility questionnaire. The intervention was rated overall with M = 3.2 out of 4 points (SD = 0.50). The trainers (M = 3.64, SD = 0.43) and the general conditions (M = 3.50, SD = 0.37) were rated best, and the training content (M = 3.04, SD = 0.50) was also positive. On the other hand, expectations regarding potential benefits (M = 2.5, SD = 3.41) were mixed. Two employees answered the open question, of which one person was “positively surprised” that they had fun and learning success. The second person would have preferred a longer training.

## Discussion

This study tested the research hypothesis whether the frequency of preventable adverse events (pAEs) would decrease in the year following the implementation of a communication training addressing health care workers in obstetrics. Overall, 2,865 of all 3,351 births in the year 2018 were analyzed as reference year and 2,846 of all 3,302 births in the year 2020 after the training. A significant reduction in pAE was observed in terms of 13.35% in the year 2018 vs. 8.83% in 2020. Subjective ratings were in favor of the effectiveness of the intervention but as there was no active control groups or a randomized control trail, the effectiveness can only be concluded from this pre-experimental design. For the reduction in pAE, there are several background variations that could account for the reduction in pAE. Future research is needed to validate the findings accordingly.

One should keep in mind that AEs in the treatment of patients will not be completely avoidable despite technical progress and sufficient training. Within the medical disciplines, obstetrics plays a special role. In the case of complications, fatal and possibly life-determining consequences can occur for mother and child, although the expectant mother is initially not a patient, and giving birth is a physical process and not a disease requiring treatment. However, the rise in maternal risk factors has increased the challenges for obstetric staff, who are additionally burdened by medico-legal aspects with a simultaneous increase in work pressure and reduced staff.

AEs, responsible for morbidity and mortality in hospitalized patients, are reported to be between 3 and 17%, and up to 50% are considered preventable [3]. In obstetrics, an incidence of up to 5.9% is reported [10]. In statistics, obstetrics is often not evaluated separately but subsumed under the specialist discipline of gynecology, which makes it difficult to state the exact number of AEs in obstetrics. Studies that explicitly consider obstetrics give incidences between 0.4% and 3.6% with a preventability of more than 50% [10, 22]. In our evaluation, the incidence of AEs is 23% and 29%, respectively. However, with the help of a very extensive trigger list, we did not only filter for the typical sentinel events and thus carried out a differentiated evaluation of 56 triggers. The evaluation according to preventability showed similar results as in the existing literature, here the incidence was 3% and 2.6%, respectively.

However, in many studies, the data are evaluated retrospectively from different documentation systems, which in turn leads to difficult comparability and distortions. Often, the mostly complex and situational conditions in the documentation systems can neither be identified nor precisely assigned retrospectively.

One method for standardized retrospective analysis of AEs is the "Harvard method" and the "Global Trigger Tool (GTT)". In a two-stage procedure, they retrospectively evaluated patient files for defined medical treatment errors. An additional 6-point scale was used to classify the preventability of the adverse events [23]. In a retrospective analysis of 311 births, 38 (12.2%) AEs were identified using the GTT. Of these, 28 (73.7%) were classified as preventable. Most of the events were grade 3/4 perineal tears, bladder voiding disorders, and anesthesia-related complications. In addition, a distinction was made between prolonged hospitalization (63.2%) and temporary harm (31.6%) [10].

Another tool for standardized recording and comparability of adverse events is the Adverse Outcome Index (AOI) [24]. This includes 10 items of obstetric adverse events, so-called sentinel events. However, the AOI does not allow differentiation between preventable and unavoidable adverse events. It also lacks adjustment for pre-existing risk factors of the patient.

One paper that used this index to measure the effect of teamwork training failed to achieve the study objective of reducing the overall number of adverse events. Only the decision to deliver time in case of an emergency section was significantly reduced [25]. Another study also used the AOI as a measurement tool to demonstrate the effectiveness of team training. Here, the score was only used with 5 parameters as a measurement tool for the primary outcome. There was also no effect on the AOI demonstrated by the training. Overall, an adverse event according to the AOI 5 was identified in 11.3% of cases [26]. However, pAEs do not always result from medical errors and can therefore not only be measured by scores like the AOI that records only sentinel events. Often, they can be traced back to so-called human factors such as insufficient communication [3, 9]. The human factor can impact patient safety via so-called “non-technical skills” (NTS). NTS consist of a variety of cognitive skills (e.g., situational awareness), social skills (e.g., ability to work in a team), behavioral skills (e.g., effective communication), and personal skills (e.g., individual stress management). Especially cognitive skills are connected to patient safety incidents because they are related to impaired situational management or delayed treatment decision [27]. Another practical example in which the human factor of both medical staff and obstetric patients can lead to pAE, are language barriers. Language barriers can lead to the poor comprehension of diagnosis and treatment options, thus affecting effective information flow and decision-making [28]. Patient safety can therefore be improved by strengthening the human factors and addressing language barriers. One point of action could be respecting the patient perspective by for example involving patient representatives. An essential contribution to this is the teaching of clear and structured communication [29].

The present study shows a statistically significant reduction in the total number of pAEs after a communication training was introduced. The communication training was substantially more focused on communication than on teamwork compared to other interventions. In most other interventions, teamwork training is implemented; but it requires sufficient preparation and resources at the site, such as time capacity or operating rooms [30]. In the categories of diagnostic errors and birth position, on which clear and structured communication has a great influence, a significant reduction could be achieved. These aspects were specifically trained in the communication lessons and the subsequent microteaching sessions. The training contents of more structured handovers with the ISBAR strategy as well as the introduction of the closed-loop communication technique were intended to contribute to the avoidance of errors and ambiguities. The closed-loop technique, which was developed in emergency medicine, shows small to medium effects in the literature [31, 32], where it was mostly evaluated in the context of broader training on teamwork in critical situations [31–33]. The training sessions in the TeamBaby project focused on a short intervention aiming at communication and behavior change. Afterwards, healthcare workers perceived a lower risk of potential triggers for pAEs.

Another important aspect of the training was the role of hierarchies. Role-plays were used to practice giving timely warnings of possible avoidable mistakes in critical situations, regardless of the professional and hierarchical position of the person acting. The so-called "Speaking-Up" should help to overcome hierarchical levels to address safety-relevant concerns and thus increase patient safety [34]. The effectiveness of trainings using Speaking-Up has been demonstrated in some studies, but the results are mixed [35]. The increase in self-efficacy beliefs in difficult situations in the present study is an indication of the effect of Speaking Up training to object to those superior in hierarchy.

In general, staff reported that the training increased their awareness of safe and effective communication. The general awareness of the role of communication was strengthened in the training using an educational film [36]. Positive effects of the training may therefore also be because the awareness of possible negative effects of communication and the awareness of one's role improved. A conceivable indication here is the result, paradoxical at first glance (marginally significant), that the staff's assessment of their general self-efficacy decreased after the training. It is possible that the training has increased the sensitivity for the susceptibility to errors in communication so that the participants assess their communication skills more critically than before the training as demonstrated by the self-reported data. Although these results indicate the overall effectiveness of the training and explain potential mechanisms, it must be borne in mind that the hypothesis, namely that the reduction of pAE can be achieved by improving interdisciplinary communication, cannot be answered without alternative explanations. Due to the large population and the time passed between 2018 and 2020, a specific reduction in pAE cannot be attributed to the intervention. It is possible that changes in the background or a heightened awareness for patient safety risks accounted for the change in pAE.

An extensive review of the effectiveness of communication training in obstetrics examined 71 studies, which showed large differences in the quality and design of communication training and studies [16]. In most cases, broader teamwork training with a focus on communication was implemented and had an impact on proximal outcomes such as communication skills or behavior. In total, however, only three studies looked at the direct impact on patient safety. In two of these three studies, a positive effect of communication training on patient safety could be demonstrated. However, the constructs examined were still very heterogeneous; only one study used the AOI as an outcome parameter. Especially in the comparison of these three studies, it can be stated that both the concrete and change-sensitive identification and recording of (avoidable) adverse events are of high value, as well as training aimed at concrete behavioral changes. If only very serious adverse events ("sentinel events") or insufficient risk factors are collected to determine the preventability of corresponding events, positive effects of communication training may not be identified. On the other hand, it is also conceivable that an intervention was not sufficiently theory-based or comprehensive to achieve positive effects. Accordingly, both the interventions and the data evaluation should be planned in an interdisciplinary team with the stakeholders on-site and adapted to the circumstances.

To reduce pAEs through training, it is particularly important to understand their key active components making the training work. As early as the 1990s, a key role was attributed to the human factors. Even though AEs caused by human factors will never be 100% preventable, effective risk management should always include communication training to strengthen the human factors in the team [5]. However, it seems to be the combination of different aspects, but further research is needed to test this in more detail.

In the future, in addition to strengthening human factors, it may also be useful to include addressing risk factors in a structured risk management. In the present work, risk factors were filtered in the cases of an AE. If risk factors are differentiated into those that can be controlled and those that cannot be or controlled, this can be included in individualized risk management. In this way, risk factors that can be controlled can be clearly communicated to the team and appropriate prophylactic measures can be taken. Checklists, adapted for the most important obstetric risk factors, are useful tools for risk-conscious individual birth management [37].

Limitations must be considered when interpreting the present results. In the evaluation of the individual categories of pAEs, no statistically significant reductions were found in peripartum therapy delay. This can be explained, among other reasons, by an insufficient differentiation of the categories so that our data cannot reflect pAE according to whether they occurred before, during or after birth. In the future, it may be useful to differentiate these categories into antepartum, peripartum, and postpartum birth management. In addition, management of special obstetric emergencies such as emergency cesarean section, shoulder dystocia, or postpartum hemorrhage should be considered. There was no statistically significant improvement in inadequate fetal monitoring, indicating that not all pAE improved over the course of the analysis. However, this does not impact the study findings negatively because effects of improvement in this category are more likely to come from special training that teaches the interpretation of CTGs during labor than from general communication training [38].

The main limitation is the lack of a control group. A pre-post comparison of the period after the training without the possibility of controlling the results for the communication training was conducted. Therefore, it cannot be ruled out that the reduction in pAEs is at least partly due to the further development in obstetric care or to other influences for which no controls could be conducted. It is possible that a heightened awareness had an unsystematic impact on pAE instead of systematic training effects. Moreover, the background variations must be considered, as the sample size comprises a large number of cases and this complicates the specific evaluation of the reduction in pAEs. In addition, the impact of the COVID-19 pandemic must be considered. Theoretically, the training helped to mitigate the negative effects of the pandemic, but we also do not have a comparator with this regard to test for the mechanisms. Accordingly, we cannot link the reduction of pAE to the intervention without alternative explanations and future research is needed to establish whether communication trainings can actively reduce pAE.

In addition, only half of the training participants completed the questionnaires in a form that could be evaluated. Thus, the evaluation results must be interpreted with caution due to the potentially selective drop-out and lower power. It is possible that mostly HCW who experienced the intervention as helpful answered the questionnaire at the second time point while the more critical ones did not. The hospital where the intervention and analyses were conducted is a teaching hospital with a corresponding high staff turnover. In the period between the measurements before and after the intervention, people may have left the obstetrics department and accordingly were no longer available to respond. In collecting the data, the present study used retrospective routine data that can be extracted and analyzed at any time point. In addition, the conditions for pAEs, especially the communication aspect, cannot always be retrieved from the available documentation systems with perfect validity and reliability. This reduces the quality and consequently the internal validity of the extracted routine data. Thus, this opens many further questions, which should be researched in the future.

## Conclusions

Human error in medicine should be addressed constructively by communication training. The occurrence of adverse and preventable adverse events needs to be handled more transparently to increase preventive efforts. Observational studies in which a differentiated categorization into adverse and preventable adverse events is carried out by external experts immediately after the occurrence of an event could be a useful approach for further studies. This study demonstrated such a categorization approach which should be implemented in practice and used for research. Future trainings can make use of this categorization approach [12] but also the training used in this study [21].

In general, it seems beneficial to design communication trainings in a form that focuses on medical emergency situations but also on modifiable human factors to overcome them. Here, the optimization of communication plays a key role. This study is a first indicator that communication trainings, among other factors, can help to reduce pAEs and thus increase patient safety and an improved satisfaction. Therefore, communication tools should be integrated into interprofessional training alongside medical emergency training in obstetrics. This also can help to avoid the risk of becoming a second victim [14]. Communication training strengthens resilience and improves teamwork [39] and can benefit all involved parties.

## Data Availability

The datasets generated during and analyzed during the current study are not publicly available due to evaluation of the data for further analyses but are available from the corresponding author on reasonable request.

## Appendix 1

List of extracted criteria regarding adverse events; categories, thresholds, and filters for adverse events (AE)

Table of the training contents

**Table:**

Category	Adverse Event (AE)	Definition/Further Operationalization
Maternal	Allergy	
	Anemia	Hb < 8 mg/dL
	Postpartum length of stay	> 3 days after vaginal birth
	Blood loss	> 1000 mL
	Diabetic ketoacidosis	
	Disseminated intravascular coagulation (DIC)	
	Eclampsia	
	Electrolyte derailment	
	Fever	> 38.5 °C
	Labor arrest	Caesarean section necessary
	Hypertension	> 180/110 mmHg
	Hypotension	< 90/60 mmHg
	Infection	Treatment with antibiotics
	Intubation*	
	Seizures	
	Manual placenta detachment	Non-delivered placenta
	Placental tissue after cesarean section	Curettage necessary
	Third degree laceration	
	Fourth degree laceration	
	Other laceration	Vaginal, perineal, labial
	Thyroid crisis	
	Death	
	Precipitate delivery	
	Unrecognized maternal disease	
	Unexpected re-admission	
	Uterine rupture	
	Prolonged second stage	> 120 min
	Transmission to intensive care unit*	
	Placental abruption	
	Wound healing disorder	
Fetal	Near-SIDS	Near Sudden Infant Death Syndrome
	APGAR	1 min APGAR < 7
	Acidosis	Cord pH < 7.1 or base excess < − 12
	Bradycardia	FHF < 60
	Birth trauma	Fracture
	Seizures	
	Meconium aspiration	
	Umbilical cord prolapses	
	Death	
	Shoulder dystocia	
	Unplanned admission to intensive care unit*	
Interventional	Transfusion	
	Failed anesthesia	
	Failed instrumental vaginal delivery	Caesarean section necessary
	Failed induction of labor	Caesarean section necessary
	Communication problem	
	Emergency hysterectomy	
	Emergency caesarean section	
	Unplanned caesarean section	
	Use of more than 1 instrument in vaginal delivery	
	Delayed intervention in case of pathological CTG	Decision-delivery time > 30 min
	Delayed intervention in case of postpartum hemorrhage (PPH)	
	Caesarean section on request	No medical indication
Organizational	Incomplete documentation	
	Medication errors	
	Communication problems	

*In the hospital where the study was conducted, women are frequently transferred to the ICU before or after delivery without the need for intubation, for example, in the case of severe hemolysis, elevated liver enzyme levels, low platelet count (HELLP syndrome).

**Table:**

Duration in minutes	Content/ exercise	Description	Goal and transfer
30	Introduction round	Introduction of the project and trainers. The participants choose a picture card, introduce themselves and describe why this card represents a good childbirth/ communication	Create an open atmosphere, illustrate differences in expectations towards safe births
20	Short film “Just a routine operation”	Participants are asked to look for communication deficits in the film, followed by a moderated discussion on mistakes and possible solutions introducing the “10-for-10” strategy	Enhance example-based learning, strengthen awareness of the role of team communication, illustrate communication failures, introduce the “10-for-10” rule
20	Exercise “Tangram”	Two participants are sitting with their backs to each other, and one participant gives instructions on how to place Tangram tiles to form a figure that only they can see. The other participant arranges the tiles according to the instructions. In the end, the resulting form is compared to the template. In the first round, the second participant is not allowed to ask questions, in the second, they are. After the task, the trainers discuss the “closed-loop communication” strategy	Understanding that simply “correct” communication is not sufficient, train clear team communication (“closed-loop communication”)
15	Active break	Participants are asked to find similarities with each other, the person with the most similarities to others gets a reward (chocolate)	Positive experience and identity
40	Exercise “Empathy maps”	Each group creates an „empathy map “ for the other occupational group/ expectant mothers. Questions: What are their tasks/ feelings/ needs/ fears? In a discussion, the trainers point out common misunderstandings and similarities between groups	Change of perspective, taking different roles, recognize outcomes of own actions
35	Exercise “Hand-over”	An unstructured hand-over is given to a first participant. This person repeats the hand-over to a second participant who was waiting outside the door. This participant gives a final recall, followed by a moderated discussion if and how participants structured their hand-over	Introduce the team communication structure “ISBAR” (Introduction, Situation, Background, Assessment, Recommendation), sufficiency of information
10–15	Exercise “Interpersonal adaption”	Participants are asked to inform a stressed expectant mother (trainer) about a concerning diagnosis	Communication with different recipients, simplify language
10–15	Exercise “Speaking-Up”	The trainers present a study on how few HCW spoke up in a simulated setting where a patient’s life was endangered, followed by a discussion of a typical obstetric situation and roleplay to practice speaking-up. Handout card “Speaking-Up”	Overcome hierarchies in teams and learn to voice patient safety concerns
10–15	Discussion “Stop-Inject”	Participants are asked whether they have had medication errors before, before they are invited to discuss strategies on how to use communication to avoid medication errors	Avoid medication errors through safe communication
5	Wrap-up	The trainers summarize the training contents	Recap
30	Behaviour planning intervention	Participants are given the behaviour planning intervention sheet. They are asked to identify a key component/ aspect of communication they want to work on independently and fill in the sheet	Identify goals for individual learning after the training, transfer to everyday work life, maintenance
10	Short evaluation	Participants are handed the feasibility questionnaire; a short feedback round is completed	

Table with the training contents.

## Abbreviations

AE: Adverse event
pAE: Preventable adverse event
HCW: Health care workers
AOI: Adverse outcome index
CTG: Cardiotocography
ISBAR: Identify, Situation, Background, Assessment and Recommendation
LGA: Large for gestational age
GTT: Global trigger tool
IOL: Induction of labor
CS: Cesarean section
PROM: Premature rupture of membranes
